# Public priorities for osteoporosis and fracture research: results from a focus group study

**DOI:** 10.1007/s11657-020-00766-9

**Published:** 2020-06-16

**Authors:** Ashley Hawarden, Clare Jinks, Waheed Mahmood, Laurna Bullock, Steven Blackburn, Stephen Gwilym, Zoe Paskins

**Affiliations:** 1 Primary Care Centre Versus Arthritis, School of Primary, Community and Social Care, Keele University, Stoke-on-Trent, ST5 5BG UK; 2 Haywood Academic Rheumatology Centre, Midlands Partnership NHS Foundation Trust, Stoke-on-Trent, ST6 7AG UK; 3 Oxford Trauma, Nuffield Department of Orthopaedics, Rheumatology and Musculoskeletal Sciences, University of Oxford, Oxford, UK

**Keywords:** Osteoporosis, Research priorities, Focus groups, Patient and public involvement

## Abstract

**Summary:**

Four focus groups were conducted with members of the public to identify important areas for future osteoporosis research. Participants identified priorities to increase public awareness of osteoporosis, reduce delays in diagnosis, improve communication between healthcare providers and to improve follow-up and information provision about causes of osteoporosis, medication harms and prognosis.

**Purpose:**

Patients and the public must be involved in setting research agendas to ensure relevant and impactful questions are prioritised. This study aimed to understand what people living with osteoporosis and fragility fractures felt was important to research, to inform the content of a national survey on research priorities in this area.

**Methods:**

Focus groups were conducted with members of the public with experience of osteoporosis or fragility fractures. The topic guide was co-developed with a patient and public involvement research user group, and explored participants’ experiences of osteoporosis including diagnosis, management and effect upon their lives, what aspects of their ongoing care was most important to them and what about their care or condition could be improved. Focus groups were audio-recorded, transcribed and analysed thematically.

**Results:**

A total of twenty-three participants were recruited to four focus groups. Analysis identified two main themes: challenges in living with osteoporosis and healthcare services for osteoporosis. Information needs was a further cross-cutting theme. Participants called for increased public awareness of osteoporosis and wanted healthcare services to address conflicting messages about diet, exercise and medication. Participants described long delays in diagnosis, poor communication between primary and secondary care and the need for structured follow-up as important areas for future research to address.

**Conclusion:**

The findings from this study provide an understanding of research priorities from the perspective of patients and the public, have informed the content of a national survey and have implications for patient education, health services research and policy.

## Introduction

Worldwide, osteoporosis accounts for approximately 9 million fractures annually [1]. As life expectancy rises, the global incidence of osteoporotic fractures is predicted to increase. Low trauma fractures are associated with reduced physical function, impaired mobility, loss of independence and increased mortality risk [2], costing the UK an estimated £4.4 billion per annum [3].

Evidence-based treatments are recommended by UK guidelines for patients with osteoporosis (underlying bone fragility) and/or high fracture risk [4]. However, 49% of women do not receive pharmacological treatment following a fragility fracture. This osteoporosis care gap has been described by experts as the “osteoporosis crisis” [5–7].

It is clear that research to improve the identification, management and support of those at risk has never been more important. However, to address the crisis that patients with osteoporosis are facing, there are many possible research agendas to pursue. Traditionally, health research priorities have been identified by researchers and stakeholders. However, patient and public involvement in research, including the prioritisation of research agendas, is now well established [8–12]. Involving patients and the public ensures that research is grounded in patient relevance, research questions are meaningful and important research topics are identified that researchers may not have previously considered [15].

There is a lack of research into the priorities of people with experience of osteoporosis. The aim of this exploratory study was to elicit, for the first time, the research priorities of people living with osteoporosis and fractures in order to inform the content for a national survey on research priorities [13]. Secondly, we report patient and public involvement and engagement (PPIE) in this priority setting research.

## Methods

Focus groups facilitate group interaction, allowing participants to “spark” ideas from each other, supporting the generation of research questions, ideas and priorities [14]. For this reason, focus groups were selected as the most appropriate method to meet the aims of this study, by supporting participants to discuss broad areas of research priority through group interaction, and as used in previous priority setting research [15, 16].

### Participants and recruitment

Inclusion criteria were deliberately non-restrictive to enable people with a wide range of life experience to participate and facilitate breadth in our findings. Participants with any experience of osteoporosis or fracture (e.g. personal experience or being a carer or spouse/partner of someone affected) were eligible. Participants were not eligible if they were unable to give informed consent. We aimed to conduct 4–5 focus groups—a similar number to previous priority setting studies [15, 16]—which we hypothesised would be sufficient for data saturation. Focus group participants were recruited from three sources.

First, 20 individuals attending a face-to-face patient education meeting hosted by the Royal Osteoporosis Society (formerly, and at the time of study, the National Osteoporosis Society (NOS)) in a community setting in Stoke-on-Trent were invited to participate. Attendees had been invited to attend the meeting by the NOS or their local primary care practice. Attendees were given verbal information about the study and written participant information. Second, mailed invitations were posted to a regional mailing list of 85 supporters of the NOS in North Staffordshire. Third, participants (aged 50 and over with a recent fragility fracture) from an existing research cohort in Oxford, who had consented to contact about other research studies, were invited to participate. A total of 150 participants from this cohort were purposively selected (to represent a range of age and genders) to receive the mailed invitation and with the expectation of a 10% response rate.

Individuals expressing an interest returned a reply slip and were subsequently contacted to make further arrangements to attend a focus group.

Approval for the study was granted by Keele University Ethical Review Committee (ref ERP1249).

### Patient and public involvement and engagement

Patient and public involvement and engagement (PPIE) members, described as “public contributors”, from an existing group at Keele University, which has a track record in integrating and sustaining PPIE throughout the research cycle [17], were involved as research partners throughout the study. The group consists of women and men with osteoporosis, or an interest in the condition e.g. caring for a family member. Two group discussions were convened. The first discussion focused on participant recruitment, information sheets and topic guides. The second group discussion, convened after the focus groups, were completed, concerned analysis, interpretation of the results and design of the subsequent national survey. PPIE has been reported with reference to the GRIPP2 criteria [18].

### Procedures and data collection

The focus groups were conducted by two experienced qualitative researchers (a rheumatologist (ZP) and social scientist (CJ)) in October 2015. After giving full informed written consent, participants initially completed a brief questionnaire about their age, gender and experience of osteoporosis and/or fracture.

A semi-structured topic guide was co-developed with public contributors [19]. The topic guide included questions about participants’ experience of osteoporosis, including diagnosis, management and the influence of the disease upon their lives, what people thought was important in their management and what was missing or could be improved in relation to their care. Public contributors made changes that included the wording of questions, the order in which questions were asked, and suggested alternative ways of describing “research priorities”, which was felt to be a confusing term. Additionally, the group suggested adding a prioritisation exercise, by asking participants to write on a Post-it® Note the topic(s) that were most important to them towards the end of the focus group. This exercise also facilitated real-time participant validation.

### Data analysis

Focus groups were audio-recorded, transcribed verbatim and subsequently imported into NVivo 11 to facilitate analysis. Thematic analysis was predominantly conducted by WM and AH, following standardised qualitative methods [20]. Transcripts were read and re-read to facilitate data familiarisation. Participants’ responses were then coded and categorised into preliminary themes and subthemes. These were reviewed and further refined before researchers met to determine agreement. Initial themes were discussed between the multidisciplinary team (AH, WM, CJ and ZP) and a coding framework agreed. Following the initial analysis, two overarching themes (living with osteoporosis and treating osteoporosis), with 30 total subthemes were used to inform draft survey development. Researchers again met with the public contributors to present each subtheme along with example quotations. The group discussion identified 10 new themes previously overlooked, and a further cross-cutting theme relating to information needs, resulting in revision of the coding framework and further iterative analysis. Post-it® Note written comments were transcribed, mapped onto the coding framework and also presented as a simple count.

## Results

**Fig. 1:**
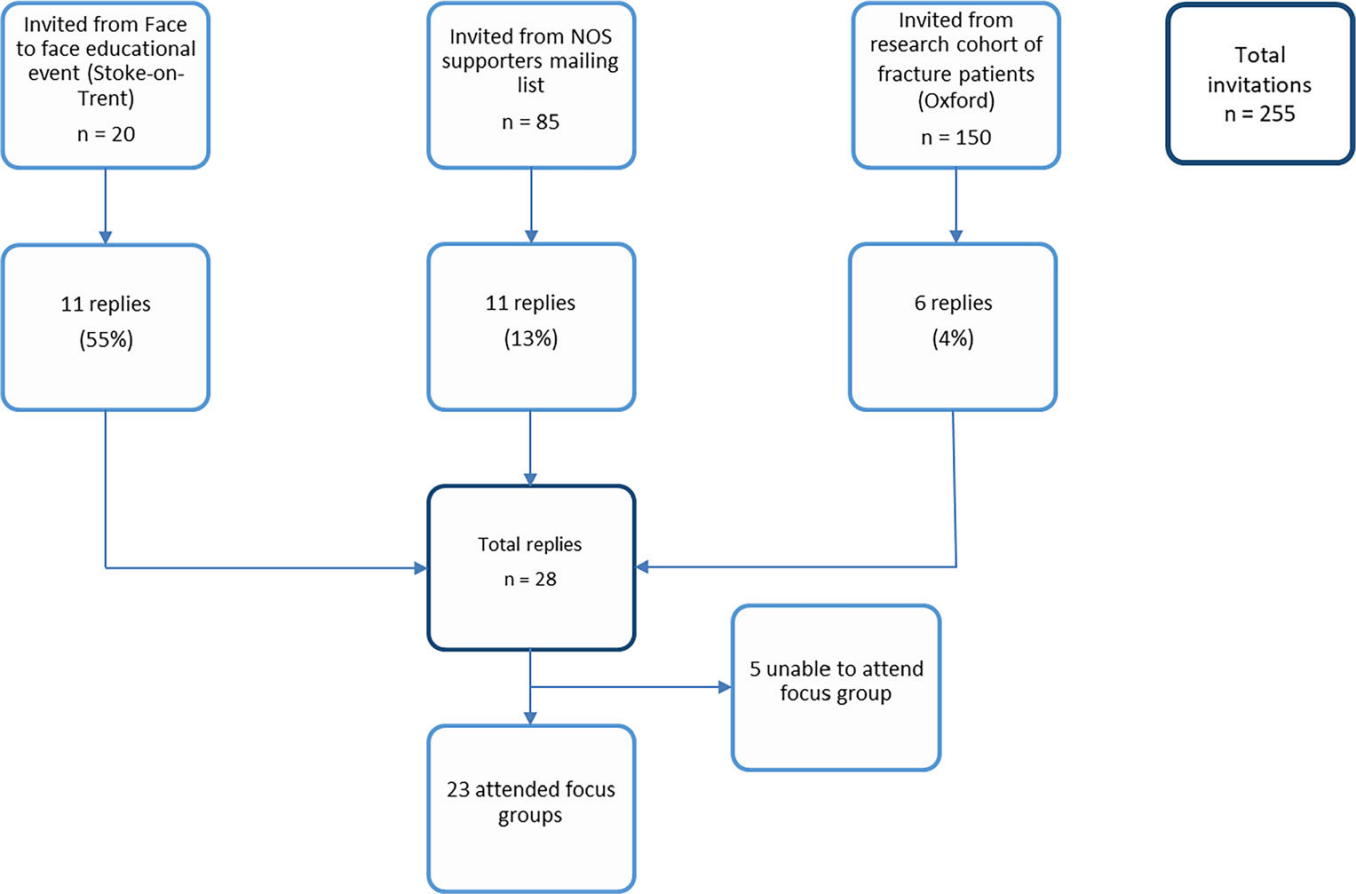
Participant recruitment

Twenty-eight of the 255 (11%) individuals invited expressed an interest in participation, with 23 of these being able to attend one of the focus group dates offered (Fig. 1). Four focus groups were conducted (three in Staffordshire, one in Oxfordshire) and the demographics of participants are detailed in Table 1. In summary, the participants consisted of 18 females and five males. A total of 19 participants reported having a diagnosis of osteoporosis and 14 reported sustaining a previous fragility fracture.

**Table 1:** Characteristics of participants

Sex	Male	5 (22%)
	Female	18 (78%)
Age (years)	< 50	1 (4%)
	50–59	2 (9%)
	60–69	9 (39%)
	70–79	10 (43%)
	80 +	1 (4%)
Self-reported diagnosis of osteoporosis		19 (83%)
Self-reported previous fracture		14 (61%)
Fracture site	Hip/femur	4
	Wrist	6
	Spine	4
	Other	9

Challenges in living with osteoporosis encompass subthemes relating to participants making sense of their condition, understanding causes and prognosis, discussing their condition with others and the effect on daily life. The theme relating to healthcare services encompasses subthemes relating to participants understanding of diagnosis, long-term management and follow-up. Information needs was a further cross-cutting theme that spanned both living with the condition and healthcare services. Within each subtheme, participants described their main areas of concern and suggested potential solutions.

### Challenges in living with osteoporosis

#### Understanding causes

In general, participants understood that post-menopausal women were at risk of developing the disease.

Participants wanted to understand associations of osteoporosis with other medical conditions. In particular, this included thyroid disease, diabetes and malabsorption. Some participants recognised that bone health could not be explained entirely by lifestyle and wanted an understanding of bone physiology and genetic factors:

“…. really interesting to know whether there is any link with a genetic or family history and to whether there is a solution to that.” (FG4:P3)

#### Effect on day-to-day life

Participants did not feel prepared for the effect a diagnosis of osteoporosis would have upon their lives.

“I was shocked; I was quite upset about it and I didn’t know how to live my life after that.” (FG3:P1)

Participants discussed how osteoporosis affected their activities of daily living, including self-imposed restrictions in mobilisation due to the fear of falling and subsequent fracture. Participants emphasised the effect adverse weather conditions could have on their ability to safely mobilise. Participants who were in pain due to previous fractures also identified a need for advice on day-to-day activities (e.g. putting on a bra) which they found difficult.

To help individuals cope with the emotional and physical consequences of osteoporosis, participants suggested greater access was needed to psychological and peer support (e.g. community groups) and that healthcare professionals needed to be more aware of the psychological aspects of the disease.

#### Awareness

Participants perceived the general population to lack understanding of osteoporosis and why osteoporosis is important. This caused participants difficulty when attempting to talk to friends and family about their condition:

“It sounds bad, the term sounds bad; it sounds as though you’ve got some infectious disease right away and I’m sure when you say it to somebody they look at you blankly, what’s that, and how do you spell that, then? And what’s that?” (FG3:P3)

Some participants also stated that the condition was normalised by friends and family as a consequence of ageing, or viewed as insignificant when compared with other health problems. One participant described the response of a relative when seeking support from them after receiving a diagnosis of osteoporosis:

“It’s not cancer, what are you worried about?” (FG3:P1)

To improve the general population’s understanding of osteoporosis, participants suggested the need for more visible awareness campaigns. Participants also recommended that education regarding bone health and osteoporosis should be commenced at school age, with the intention of encouraging healthy behaviours to reduce the risk of future disease. At present, participants felt osteoporosis was not a disease in which preventative action was considered until it was “too late”.

#### Role of diet and exercise

Participants felt that there was an overwhelming amount of advice regarding diet in the public domain; however, participants found it difficult to interpret dietary advice that was relevant to bone health. There was particular confusion around the benefits and drawbacks of dietary supplementation with calcium, magnesium and vitamins K, B and D. Additionally, participants recognised that lifestyle advice was often contradictory. For example, advice to optimise calcium intake could be with at odds with advice to take foods low in cholesterol:

“We’re told to keep out of the sun, but vitamin D is essential. Again, for the absorption of calcium we’re still told to keep off high cholesterol foods such as cheese and so forth.” (FG3:P5)

Generally, participants had a strong desire to keep themselves active through physical activity but were again confused by contradicting advice regarding the types of exercise that were most beneficial. One participant worried that some activities could be potentially harmful:

“I couldn’t possibly do weight lifting because I’m so fragile. I would just break my wrists.” (FG4:P1)

Participants wanted clear consensus and accurate information from healthcare professionals at the time of diagnosis regarding safe exercise and optimal diet for improving bone health in osteoporosis.

#### Prognosis

Participants were unclear about the natural progression of osteoporosis. One participant explained their uncertainty regarding the future:

“I was concerned about how it was going to affect my life over the next however long I’d got. Am I going to get worse and worse and worse? ... as the years go on, how is it going to progress? I don’t know. You don’t know, do you? ... There isn’t enough known about it.” (FG2:P4)

The progressive nature of the disease and perceived limited therapeutic options underpinned some participants’ view that osteoporotic fractures were unavoidable. One participant compared osteoporosis with cancer, suggesting that cancer symptoms could be managed or cured, but with osteoporosis related fractures this was not possible:

“I’ve always got this thing that when my back goes the pain associated with it, I’ve got this worry, it’s not something you can go and get fixed, unlike cancer theoretically, to some extent you can fight that and that can possibly get fixed. You can’t with that.” (FG3:P3)

#### Information seeking

Upon diagnosis, participants expressed a desire to find out more information regarding their condition and how it may affect their day-to-day life. Participants described seeking information from a vast number of sources. This included general practitioners (GPs), specialist doctors, practice and specialist nurses, Internet, television, magazines, charities (specifically NOS), family and friends. Primarily participants preferred digital media, while recognising this may not be suitable for all. Participants valued the NOS nurse specialist helpline which they utilised both for information and emotional support. Participants suggested that these resources should be routinely signposted by healthcare professionals:

“...as soon as somebody is diagnosed with having a low bone density, it should be flagged up immediately and that person should then be given … the Osteoporosis Society’s email address or website.” (FG4:P1)

### Healthcare services for osteoporosis

#### Role of screening

In general, participants described diagnosis as a prolonged and protracted process. Even in the presence of obvious risk factors, participants explained how they needed to advocate for their own health to be taken seriously.

“I broke my elbow and my ankle and nobody mentioned osteoporosis, nobody did a scan, nobody did anything. I get very frustrated knowing that something could have been picked up then. I could have had medication so that nine years later I wouldn’t have had a vertebral fracture. It wasn’t until I had the vertebral fracture that the word osteoporosis was mentioned.” (FG1:P19)

As a result of this, participants queried whether osteoporosis was taught on the undergraduate curriculums of medical schools. Participants indicated that this could be a priority so that future doctors would be more equipped to recognise risk factors.

In the context of perceived diagnostic delay, participants discussed the opportunities for earlier identification through screening programmes. Specific suggestions included a Dual-energy X-ray Absorptiometry (DXA) screening programme or a formal osteoporosis review in a primary care well woman/man clinic. However, participants were mindful of the financial implications of such programmes.

#### Drug therapy

Participants mostly had negative perceptions of drug therapy for the management of osteoporosis. Bisphosphonates and the side effects osteonecrosis of the jaw and atypical fractures were of particular concern for participants:

“Osteonecrosis, yeah, which is an excess of jaw growth and you could end up like the Elephant Man. I think it should be highlighted a bit more because I went into it a bit lackadaisical and not thinking what the consequences would be.” (FG4:P3)

These concerns appeared to be perpetuated by the media, dentists and second-hand stories:

“I just think it’s worrying. The last time I went to see my dentist and he just said, ‘We just need to monitor your jawbone.’ … and I said, ‘I’m on a different one. I’m not on the bisphosphonates.’ And he said, ‘The jury’s still out on the rest of them.” (FG1:P1)

In order to improve confidence in taking medications for fracture prevention, participants indicated a need for more information on the mechanism of action of drugs, side effect profiles and benefits of taking fracture prevention drug treatment. Participants were particularly keen to understand how risk of fracture might improve if they took medication compared with lifestyle intervention alone. In order to alleviate concerns regarding medication, participants highlighted a need for further research into the development of new drugs with more favourable side effect profiles.

#### Follow-up

Generally, participants reported that follow-up regarding their osteoporosis did not routinely take place. However, follow-up was perceived as important to participants because it could give them insight into their disease progression:

“It doesn’t achieve anything being told you’ve got it, if you don’t actually find out whether you’re being effective in managing it.” (FG4:P2)

Participants recognised the general difficulty of monitoring the condition and uncertainty about whether treatment (with a bisphosphonate) was effective or not:

“Okay, take these.’ You’re like in no-man’s land. Is it doing anything? Is it doing me good? Is it doing me ill? Can I do anymore? And those are the questions they won’t answer.” (FG1:P3)

Participants considered that follow-up DXA would provide evidence of improvement, stability or deterioration of disease. However, some participants described frustration in obtaining a DXA and again reported having to take control of their own disease management:

“Unless I request, for example, a DXA scan repeat, as I have done, there is no on-going review. You have to, I have to go to my GP to discuss this.” (FG3:P1)

To improve satisfaction with follow-up, participants recommended a defined protocol in order to standardise care which detailed the recommended frequency of DXA.

#### Information needs

Participants had a desire to talk to healthcare professionals about their condition but found this could be difficult:

“Being able to talk about so many issues today has helped my feeling of wellbeing. It would be good to have the opportunity to talk to a GP about such matters.” (Post-it® Note: 6)

Participants described multiple examples of dissatisfaction with the information they had received, and felt that medical professionals sometimes had negative attitudes towards osteoporosis or did not take the condition seriously. There was a perception that GPs took little interest in osteoporosis. One participant described trying to open a dialogue with their GP regarding concerns they had about their medication but this was dismissed:

“I went to my GP and said ‘what do you think? There is this, this and this drug I’m on, alendronic acid. Shall I try this one?’ No, no, they’re all the same. And that’s as far as I got.” (FG3:P1)

Similarly, the same participant described enquiring about the results of their DXA:

“I asked various questions, what’s this thing about a T-score?’ Oh, don’t bother about that; it’s nothing to do with you. But actually it was! ... I just felt alone in this and I had to make my own enquiries, my own decisions and so on. And I still feel like that.” (FG3:P1)

Participants wondered if the apparent reluctance of GPs to discuss osteoporosis was in part due to a lack of knowledge:

“He knew absolutely nothing at all about osteoporosis and he admitted to me he knew nothing. He said ‘you’ve told me what I know’ and I knew nothing so I came away very shocked...” (FG3:P4)

#### Interaction between primary and secondary care

Participants reported that osteoporosis was not a priority to primary care clinicians, who were generally reluctant to prescribe therapies recommended in secondary care:

“The only way it fell to bits was between the consultant’s letter and the GP because then it’s like fight to the death to get the treatment that the consultant has recommended.” (FG2:P3)

Furthermore, participants also described a reluctance of GPs to refer them on for specialist advice:

“Refused to give me a referral to the [hospital]. So as I say, then quite a few months later my husband took me back to the doctors. She still refused to give me strontium and we’d reached stalemate so I said ‘I must have a referral’. I went back there and the doctor I saw, she said ‘oh, looking at your notes, you should have come back here six months ago’ [laughs] I said ‘I know, but I wasn’t referred. She wouldn’t refer me.” (FG3:P1)

#### Fracture identification and management

Several participants within the focus groups described sustaining fractures as a result of their osteoporosis. Participants raised concern regarding the detection of fractures (especially vertebral fractures). Participants recalled visiting healthcare professionals numerous times with pain before the diagnosis of vertebral fracture was considered:

“When I first had the first fracture, I’m afraid I was fobbed off; this is the case I hear quite often, that it’s wear and tear and it went on and on and I begged for an X-ray in the end, I was in such pain, and of course then they discovered I’d fractured my spine.” (FG1:P13)

The busy outpatient environment, viewed as chaotic and stressful was felt to influence a lack of consideration for the effect fractures can have on functioning.

“I think maybe the fracture clinic is like the fallout from the Battle of the Somme.” (FG2:P1)

Participants described early discharge to inappropriate home environments (e.g. lack of home adaptations) and a failure to consider the practicalities of living with a fracture (e.g. ability to eat and drink when in plaster). Face-to-face physiotherapy sessions, in particular, group sessions were valued; however, some participants felt that they were discharged from physiotherapy services too early. Exercise sheets to complete at home were described as less motivating and participants described difficulty in determining if they were performing exercises correctly.

### Prioritisation exercise

Participants wrote between one and four points on Post-it® notes as summarised in Table 2. The participants identified follow-up, pharmacological management (specifically risk vs benefit of drugs), screening, public awareness, prevention and causes of osteoporosis, attitudes of professionals and treatment pathways as important.

**Table 2:** Areas of importance highlighted on Post-it® notes

Subtheme	Number of responses	Example comment
Follow-up	10	“There is need for a more comprehensive follow up procedure – re scans and medication.” (Post-it®: 17)
Drug therapy	5	“Drugs and their side effects.” (Post-it®:13)
Role of screening	4	“A routine test to determine the possibility of getting osteoporosis in the future and how to prevent it.” (Post-it®:21)
Awareness	4	“Lack of general awareness (public & medical).” (Post-it®: 9)
Role of diet and exercise	3	“More information to the public about prevention (through diet, lifestyle).” (Post-it®:22)
Impact on day-to-day life	3	“The importance of managing the impact that osteoporosis has on people (Physical, psychological).” (Post-it®:22)
Interaction between primary and secondary care	3	“Diversity in how individuals are treated- large variance.” (Post-it®:11)
Understanding causes	2	“Link with thyroid problem.” (Post-it®: 2)

## Discussion

To our knowledge, this is the first qualitative study aimed to identify areas of research priority in osteoporosis for members of the public. While our findings relating to challenges experienced living with osteoporosis are perhaps already documented, we identified a number of previously undescribed priority areas relating to healthcare services for people with osteoporosis. Furthermore, despite the focus on healthcare services, there was also a call for research into biomedical areas such as genetics, highlighting again that participants do value research that may not have immediate pathway to patient benefit [21].

Our public contributors translated information needs to “having easy access to advice and information from health professionals” for the survey, which was subsequently rated as the number one priority (*n* = 1088) [13]. Osteoporosis is a condition for which patients describe significant uncertainties [22, 23]. Our findings identify specific information needs, particularly relating to non-pharmacological management and prognosis. The next step is identifying which of the “information needs” are known unknowns i.e. evidence exists but needs to be better disseminated and implemented, and which are genuine unknown unknowns, and research questions. For example, participants reported uncertainties regarding what constitutes safe exercise, as has been reported elsewhere [22, 24, 25]. To some extent, these unknowns may have been addressed by the recently published UK consensus statement “Strong, Steady and Straight”, which aims to summarise existing evidence and address inconsistencies in messages regarding best, safe exercise for people with osteoporosis [26] . Although this “information need” has been met for health professionals, the research agenda may now lie in how best to optimise this information for members of the public. When improving information provision, we need to consider not just quantity but quality, which in turn encompasses a range of issues relating to accessibility, acceptability, comprehensibility (including attention to health literacy), accuracy, comprehensiveness, relevance and utility [27].

We identified perceived conflicting messages relating to lifestyle messages around diet and sunlight exposure which is an important message for clinicians, providers of educational materials and policy makers to consider. Participants also discussed a perceived need for more public health campaigns to promote bone healthy behaviours. This may be a less straightforward issue to address as providing education alone is usually insufficient to meaningfully change public behaviours [28].

Our findings, and those of others, suggest that information, particularly in primary care, is difficult to obtain due to a combination of perceived lack of interest in the condition, or lack of knowledge [25, 29]. Positive relationships with healthcare professionals are important in facilitating treatment adherence [22, 29]. A study exploring the beliefs and attitudes of GPs in Australia revealed that GPs perceived osteoporosis to be less important than other prevalent conditions such as cardiovascular disease, but that they were concerned regarding their osteoporosis knowledge (particularly around drug therapy) [30]. More work is needed to explore the attitudes of GPs towards osteoporosis and its management, the reasons for the reported disconnect between secondary and primary care and the influence of any disconnect on outcomes of specialist osteoporosis services e.g. Fracture Liaison Services [31].

Our participants described a need for osteoporosis to be identified earlier so that treatment could commence before fractures occurred; “early identification” was also the 2nd rated priority in the national survey [13]. In this study, as reflected elsewhere, the diagnosis of osteoporosis was perceived to be a long drawn out process over which participants had to take control [32, 33], even in the presence of known risk factors and previous fragility fractures. In order to improve early diagnosis, participants prioritised the need for future research to explore the benefit and cost effectiveness of a screening programme on both a national and local basis. This issue has been addressed by the recent SCOOP trial [36], which was published after the focus groups took place; screening for osteoporosis is therefore another example where the research agenda may now concern dissemination and implementation. The long delays described in diagnosis of vertebral fractures highlighted by participants are worrying, given that patients with vertebral fracture are at high risk of further fractures [37]. The duration of and reasons for delay in the diagnosis of vertebral fractures are in need of further investigation, as has happened for other musculoskeletal conditions such as ankylosing spondylitis and rheumatoid arthritis [34–37].

The safety and benefit of drugs was discussed in the focus groups and rated as the 3rd highest priority in the survey [13]. Participants questioned whether drugs were working or not and the need for more effective monitoring and follow-up. Previous studies have shown this is an important disincentive for persisting with medication [38]. Similarly, our focus group priority exercise showed that participants felt follow-up was the most important area to improve. The findings also demonstrate misconceptions and fears related to rare serious harms with bisphosphonates, and the importance of other clinicians e.g. dentists, the media and patients’ wider social network in influencing these views. The research priority might concern how clinicians best communicate risks and benefits of treatment not only to patients, but also to the wider group of professionals involved in patient care, to ensure consistent messages.

This study is underpinned by strong PPIE which led to changes in research design and interpretation of findings. The theme relating to information needs would have been omitted without collaboration with PPIE during analysis, and this theme went on to be the highest rating item in our national survey. This study provides a potential framework for design of patient priority setting research with integrated PPIE. Public contributors did not all have direct experience of osteoporosis, yet still made important and insightful contributions. Public contributors felt a strong sense of ownership of this study and agreed to act as mentors/buddies to new osteoporosis PPIE members, facilitating an increase in capacity. In a subsequent discussion meeting, the public contributors advised on dissemination and advised on lay wording of research themes to be presented to the NOS. These themes were adopted as core research areas in the charity’s research strategy [39].

This focus group study is subject to limitations worthy of mention. The majority of participants were NOS supporters, which suggests that they seek out information in relation to their condition, may have high health literacy and may not therefore be generalisable to a broader population. This could explain the focus on information needs (and yet the lack of discussion of issues related to understanding of information). The inclusion of males could be considered a strength as traditionally men are under-represented in osteoporosis research, although the proportion of males was small. Our sample size was dictated by time (as we did not have sufficient time pre-survey to conduct further mailed invitation rounds) although inductive thematic saturation [40] was deemed to be reached with four focus groups. Finally, the role of the main focus group facilitator as a doctor and osteoporosis specialist may have influenced responses. For example, participants may have been reluctant to criticise care they had experienced for fear of causing offence.

## Conclusion

In summary, this exploratory study highlights the importance of PPIE when setting research priorities and provides a more in depth understanding of priorities for people living with or caring for those with osteoporosis. The findings illustrate a need for further health services research to address issues relating to delivery of care across primary and secondary care, provision of consistent information from health professionals, processes for follow-up and investigating delays in diagnoses (particularly of vertebral fractures). In addition, these findings have implications for public health messages around lifestyle and awareness of osteoporosis and bone health, which are of relevance to clinicians, policy makers and third sector organisations. The results were used to generate questions for a national survey of research priorities, and informed the research strategy of the Royal Osteoporosis Society, meaning that future research is more likely to be relevant to the needs of patients with osteoporosis.
